# Pattern of diseases and clinical outcomes in medical intensive care unit at a tertiary hospital in northeastern Tanzania: A three-year retrospective study

**DOI:** 10.1371/journal.pone.0282269

**Published:** 2023-02-24

**Authors:** Abid M. Sadiq, Kajiru G. Kilonzo

**Affiliations:** 1 Department of Internal Medicine, Kilimanjaro Christian Medical Centre, Moshi, Kilimanjaro, Tanzania; 2 Kilimanjaro Christian Medical University College, Moshi, Kilimanjaro, Tanzania; University of Botswana School of Medicine, BOTSWANA

## Abstract

**Background:**

The availability of medical intensive care unit (MICU) services is limited, which is the main obstacle to providing optimal care to critically ill patients. Describing disease patterns and clinical outcomes will help make better use of the limited resources. This retrospective study was conducted to determine the pattern and outcome of MICU admissions to aid continuous quality improvement in obstetric care.

**Materials and methods:**

This was a retrospective study conducted in a tertiary hospital in northeastern Tanzania. Data on participant characteristics were collected from patient records for all MICU admissions to identify the pattern of disease, length of stay, and clinical outcome from 1st January 2018 to 31st December 2020. Descriptive statistics were presented as frequencies, proportions, and tables. The odds ratio was generated for the relationship between MICU admission outcome and participant characteristics. A p-value <0.05 was considered statistically significant.

**Results:**

Of the 1425 patients analyzed, 780 (54.7%) were males. Most patients (61.5%) were admitted to the MICU from the emergency department. The overall mortality rate was 37.6%. Mortality was associated with being over 75 years old (OR 1.66, 95% CI 1.20–2.30, P 0.002), being transferred from the medical ward (OR 1.46, 95% CI 1.16–1.82, P 0.001), having a communicable disease (OR 2.63, 95% CI 1.98–3.50, P <0.001), and having cardiovascular disease (OR 1.46, 95% CI 1.14–1.86, P 0.002).

**Conclusion:**

The overall mortality rate in the MICU was high. Elderly patients, transfers from the medical ward, and short ICU stays were significantly associated with the poor outcome of MICU patients. Further studies are needed to better appreciate the causes underlying MICU admission outcomes.

## Introduction

Initially, the intensive care unit (ICU) was referred to as intensive nursing care, but since the mid-1950s, due to the development of medical therapy over the years, it changed to an ICU. The ICU has significantly improved the quality of care and outcomes for critically ill patients, mostly in developed countries [1]. The ability to perform continuous patient status monitoring is a key factor differentiating the ICU from the general wards in the hospital. In the ICU, data are continuously displayed, recorded, and readily accessible to all medical personnel caring for the patient. This is aimed at helping doctors and nurses monitor a patient’s vital trends and respond appropriately according to the patient’s needs [2].

Access to the ICU is a vital component of healthcare systems; hence, critically ill patients are admitted to the ICU to reduce morbidity and mortality [3]. Critically ill patients are medically challenging and may benefit from a multidisciplinary approach, but they are usually treated in the ICU, where the hospital has the highest mortality rates [4].

Mortality in the ICU is a global burden and varies globally depending on the disease pattern, infrastructure, and ICU staff. In developed countries such as the United States of America and European countries, the ICU mortality rate is low, with rates between 9.3% and 18.7%, respectively, while in less developed countries such as South American countries and Middle Eastern countries, the mortality rate is 21.7% and 26.2%, respectively [5, 6]. In sub-Saharan Africa (SSA), the mortality rate is high because the availability of ICU services is limited due to a lack of financial resources, technology, and well-trained staff. These remain major obstacles to providing optimal care to critically ill patients [7].

Describing disease patterns and clinical outcomes will help make better use of the limited resources available in developing countries like Tanzania. Therefore, we describe the admission patterns and outcomes among patients admitted to the medical intensive care unit (MICU) at KCMC. The obtained information will provide solid evidence on what should be done to improve the general direction of the MICU at KCMC.

## Materials and methods

### Study setting and design

The study was undertaken at KCMC in Northern Tanzania, which is a teaching hospital serving a population of around 15 million people, and its catchment area extends to the central and eastern zones [8]. In the medical inpatient department, the inpatient bed capacity is 96, with 5 beds allocated to the MICU. The critical care capability of the MICU is level 2. However, as per the requirements of the center, the staffing and resources available are around 50% of the government’s requirements [8], though actual reporting is not available. This was a hospital-based retrospective observational study conducted in the period between 1st January 2018 and 31st December 2020, which included patients aged over 13 years who were admitted to the MICU at KCMC. Patients aged 13 years and below were admitted to the pediatric wards and therefore excluded from the study. The study was approved by the Kilimanjaro Christian Medical Centre and the Kilimanjaro Christian Medical University College Research and Ethics Committee (no. 2479). Informed consent was not obtained as participants had either been discharged or were deceased at the point of data extraction.

### Study variables and analysis

Patient records were retrieved from the electronic medical hospital system at KCMC. The electronic system contains information on a patient that can be created, gathered, managed, and consulted by authorized medical staff within one healthcare center. The information that was collected included age, gender, date of admission into the MICU, department the patient was admitted from, diagnosis, date of discharge or death from the MICU, and duration of stay in the MICU. Data were taken at two points in each patient’s hospitalization: at the start of MICU care and at the end of MICU care. All data were fully anonymized using identification numbers to assure confidentiality. The data were collected and entered into a spreadsheet. The data were cleaned and entered into the Statistical Package for Social Science (SPSS) v26 (IBM; more information at https://www.ibm.com/analytics/spss-statistics-software). Patients with incomplete data were excluded. The outcomes were the pattern of disease, length of stay, and clinical outcomes of all patients admitted to the MICU in the defined period. Patients who were admitted within the defined period had their outcome identified, even if their stay in the MICU was beyond the defined period, particularly for patients who were admitted at the end of the study period. Descriptive statistics were used to describe and compare binary variables. Death rates were calculated, and univariate logistic regression was used to determine any association between mortality and admission characteristics. The objectives were summarized using narration, tables, and figures.

## Results

A total of 1445 patients were admitted from January 2018 to December 2020 into the MICU. Twenty patients had incomplete data; therefore, 1425 patients were included in the study (Table 1). Males (54.7%) were more frequent compared with females. The common age group was 26–50 years (38.8%), with a mean age of 49.4 years. More patients were admitted during the weekdays (61.7%) compared to the weekend.

**Table 1:** Demographic and admission characteristics of patients admitted to MICU.

Variables	Male	%	Female	%	Total	%
Age (years)						
<25	91	11.7	112	17.4	203	14.2
26–50	311	39.9	242	37.5	553	38.8
51–75	284	36.4	220	34.1	504	35.4
>75	94	12.1	71	11	165	11.6
Median (IQR)	50.0	(35–65)	46.0	(30–66)	48.0	(33–65)
Common Comorbidity						
Type 2 DM	119	15.3	91	14.1	210	14.7
Hypertension	132	16.9	77	11.9	209	14.7
Type 1 DM	45	5.8	57	8.8	102	7.2
HIV	33	4.2	27	4.2	60	4.2
Transfer to MICU from						
Emergency Department	513	65.8	363	56.3	876	61.5
Medical Ward	251	32.2	229	35.5	480	33.7
Other Ward	16	2.1	53	8.2	69	4.8
Outcome						
Transfer Out	443	56.8	394	61.1	837	58.7
Referral	10	1.3	10	1.6	20	1.4
Discharge Home	22	2.8	10	1.6	32	2.2
Death	305	39.1	231	35.8	536	37.6
LOS						
<24 hours	98	12.6	74	11.5	172	12.1
1–3 days	367	47.1	258	40	625	43.9
4–7 days	241	30.9	39.1	61	493	34.6
>7 days	74	9.5	61	9.5	135	9.5
Median (IQR)	3	(2–5)	3	(2–5)	3	(2–5)
Admission Day						
Weekday	479	61.4	399	62.1	878	61.7
Weekend	301	38.6	244	37.9	545	38.3
Total	780	54.7	645	45.3	1425	100

DM = Diabetes Mellitus; HIV = Human Immunodeficiency Virus; LOS = Length of Stay; MICU = Medical Intensive Care Unit.

The common comorbid conditions were type 2 diabetes mellitus and hypertension, at 14.7%, respectively. HIV was seen in 4.2% of the MICU admissions. Most of the admissions to the MICU (61.5%) were admitted from the emergency department or during admission, with 33.7% of transfers occurring after the patient’s admission. The overall mortality of the MICU was 37.6%. Most patients spent 1–3 days in the MICU (43.9%) with a mean length of stay (LOS) of 4.1 days (Table 1).

The most common specific diagnosis at MICU admission was esophageal varices (13.5%), followed by diabetic ketoacidosis (11.7%), heart failure (7.9%), chronic kidney disease (5.8%), and cardiogenic shock (5.5%) (Table 2). The mortality rate was high for acute leukemia (80.0%), followed by acute bacterial meningitis (77.8%), septic shock (77.4%), organophosphate poisoning (76.0%), and cardiogenic shock (73.1%).

**Table 2:** In-Hospital mortality of the leading causes of death in the MICU.

Diagnosis	Death	%	Admission	%	Mortality Rate (Deaths/100 Admissions)
Cardiogenic Shock	57	10.6	78	5.5	73.1
Septic Shock	48	9.0	62	4.4	77.4
Chronic Kidney Disease	45	8.4	82	5.8	54.9
Heart Failure	33	6.2	113	7.9	29.2
Esophageal Varices	33	6.2	192	13.5	17.2
Stroke (Hemorrhagic/Ischemic)	32	6.0	52	3.6	61.5
DKA	28	5.2	167	11.7	16.8
Respiratory Tract Infection	25	4.7	44	3.1	56.8
Organophosphate Poisoning	19	3.5	25	1.8	76.0
Acute Kidney Injury	12	2.2	31	2.2	38.7
Pulmonary Embolism	12	2.2	38	2.7	31.6
Tetanus	12	2.2	28	2.0	42.9
Gastrointestinal Hemorrhage	10	1.9	16	1.1	62.5
HHS	9	1.7	34	2.4	26.5
Acute Coronary Syndrome	8	1.5	26	1.8	30.8
Acute Leukemia	8	1.5	10	0.7	80.0
Status Epilepticus	8	1.5	37	2.6	21.6
Acute Bacterial Meningitis	7	1.3	9	0.6	77.8
Paraquat Poisoning	7	1.3	10	0.7	70.0
Malaria	6	1.1	21	1.5	28.6

DKA = Diabetic Ketoacidosis; HHS = Hyperosmolar Hyperglycemic State

The proportion of deaths among males (39.1%) was higher than that of females (35.8%). However, there were no significant sex differences in mortality (OR 1.15, P 0.202). The odds of mortality increased with age. The age group of 51–75 years had 1.46 higher odds of mortality (P <0.001). However, patients aged more than 75 years had 1.66 odds of mortality (P 0.002) (Table 3).

**Table 3:** Predictors of mortality among patients admitted to MICU.

Variables	OR	95% CI	P value
Sex			
Female	1		
Male	1.15	0.92–1.42	0.202
Age (years)			
<25	1		
25–50	0.68	0.54–0.85	<0.001
51–75	1.46	1.17–1.83	<0.001
>75	1.66	1.20–2.30	0.002
Transfer to MICU from			
Other Wards	1		
Emergency Department	0.73	0.59–0.92	0.006
Medical Ward	1.46	1.16–1.82	0.001
LOS			
>7 days	1		
4–7 days	0.27	0.20–0.34	<0.001
1–3 days	1.47	1.18–1.82	<0.001
<24 hours	10.32	6.83–15.57	<0.001
Day of Admission			
Weekday	1		
Weekend	1.11	0.90–1.39	0.332
Disease Category			
Noncommunicable Disease	1		
Communicable Disease	2.63	1.98–3.50	<0.001
Noncardiovascular Disease	1		
Cardiovascular Disease	1.46	1.14–1.86	0.002

LOS = Length of Stay; MICU = Medical Intensive Care Unit

Patients admitted from the emergency department or during admission had lesser odds of mortality (OR 0.73, P 0.006), compared with patients transferred from the medical ward, who had higher odds of mortality (OR 1.46, P 0.001) (Table 3). Patients who stayed in the MICU for <24 hours were ten times more likely to die (P <0.001) than patients who stayed for more than seven days. Patients admitted to the MICU for a communicable disease had 2.6 higher odds of mortality (P <0.001), while patients admitted for a cardiovascular disease had 1.4 higher odds of mortality (P 0.002).

**Fig 1:**
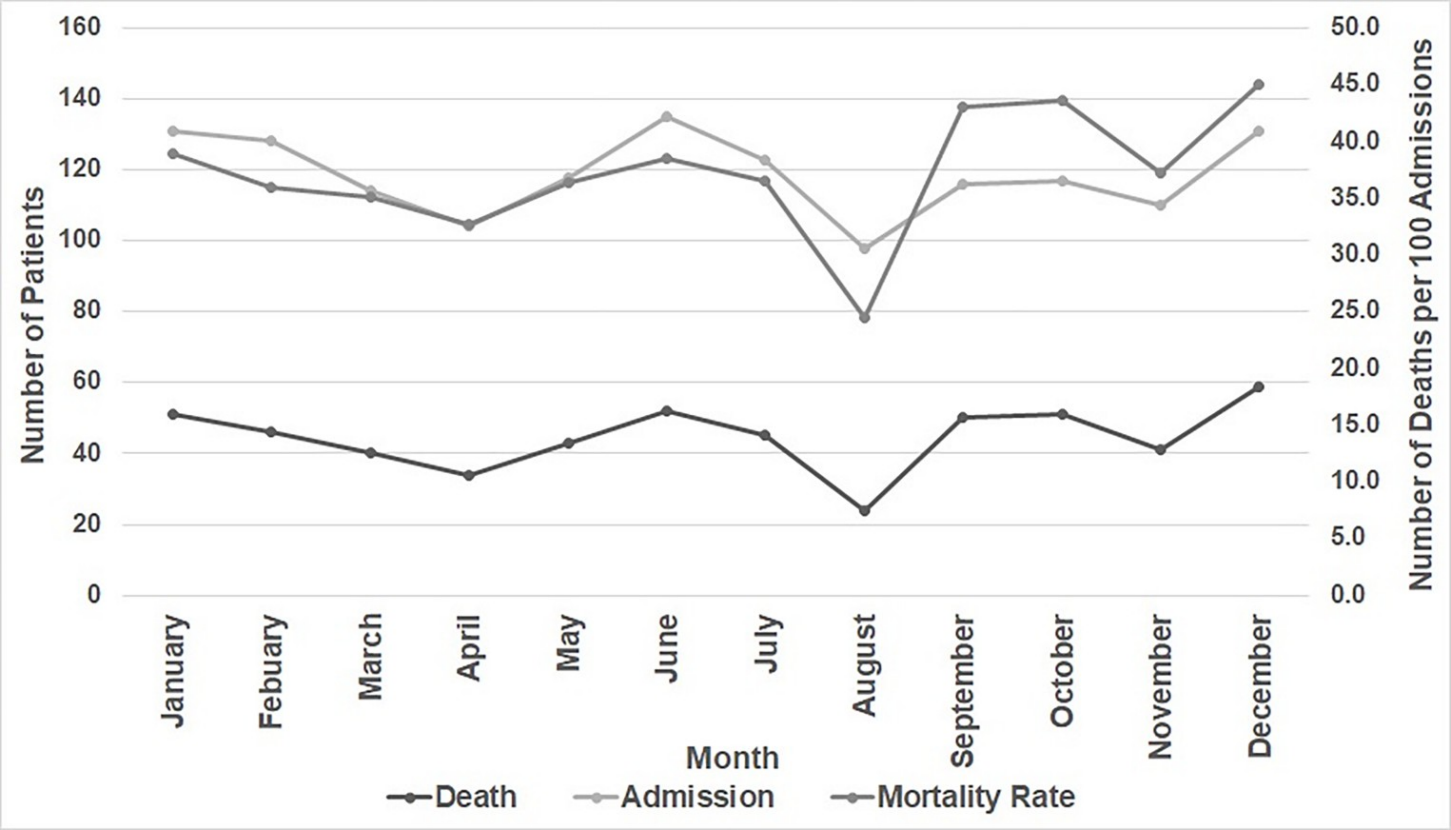
Monthly analysis of admission versus death over three years in the MICU.

The highest mortality rate (45.0%) was observed in December, followed by October (43.6%) and September (43.1%). The lowest mortality rate (24.5%) was observed in August (Fig 1).

## Discussion

The clinical outcome of patients admitted to the MICU is determined by their clinical condition at the time of admission, the level of experience and training of the staff, the ICU’s resources, infrastructure, and capacity [5, 6]. In this study, the overall mortality rate for patients in the MICU was 37.6%. This finding is comparable with another study in Tanzania (41.4%) [7]. This is also similar to the studies conducted in Nigeria (34.6%), Ethiopia (38.7%), Uganda (40.1%), and Kenya (53.6%) [6, 9–11]. However, this result was higher than studies done in developed countries such as the United States of America (5.6%), Scandinavian countries (9.1%), and Korea (13.8%) [3, 12, 13]. This difference might be due to a lack of necessary medical equipment, infrastructure, and training [6, 7].

The LOS can assess the efficacy of hospital management, quality of care, and functional evaluation. ICU LOS is determined by the cause of admission, which differs among diagnoses. Reduced LOS is associated with fewer hospital-acquired infections and medication side effects, including a reduced burden of medical fees and increased bed turnover time [14]. In this study, the median length of MICU stay was 3 days, which is relatively similar to the other African countries [6, 11]. There is a slight difference in the LOS between developed and developing countries, with the latter having an increased LOS. Because of advancements in medical therapy in the early 2010s, the mean LOS is now 3.5 days [15]. In developing countries, such as Brazil and Ethiopia, the mean LOS observed is 5 and 4.1 days, respectively [16, 17]. Patients who stayed in the MICU for less than 24 hours were 10 times more likely to die. This may not be an adequate representation since the reason they stayed for less than 24 hours is because they died. It is rare for critically ill patients to be in the ICU for less than 24 hours and be transferred out. This difference might be due to factors such as late arrival at the ICU and delay in intervention, shortage of critical emergency drugs, resuscitation ability of the MICU, and absence of airway management equipment in the emergency department [6]. However, this result was different from the study conducted in Ethiopia, where the LOS of ICU mortality was more than 14 days [18].

Disease patterns tend to vary according to age, race, lifestyle, immune status, co-morbidity, and region. The mean age in this study was 49.4 years, with male predominance. In the ICON study, low- and lower-middle-income countries tend to have a slightly younger population (55 years) compared to high-income countries (62 years) [5]. The age distribution of ICU admissions in developing countries, such as Nigeria, shows a mean age of 38.2 years with a higher male predominance [9]. The mean ages in developed countries, such as the United States of America and the United Kingdom, are 60.4 years and 57.4 years, respectively, with a slightly higher male predominance.

Over 10 years ago, the trends were different in developing countries, as can be observed in Taiwan. Although the mean age and male predominance were similar to those of developed countries, the infectious disease related causes of admission were 47.1% and cardiac-related causes were 5.6% [19]. Currently, in developing countries such as Brazil and Ethiopia, the trends are different from those of developed countries and those over from 10 years ago. In Brazil, 19.2% of ICU admissions are cardiac-related, followed by infectious diseases (18.6%) and neurological diseases (9.6%) [16]. Similarly, in Ethiopia, the ICU admission trends were observed as being cardiac (26.1%), infectious (20.3%), and neurological (19.8%) [17]. In Tanzania, infectious diseases led the admission category (19.5%), followed by cardiovascular (6%) and metabolic (6.1%) diseases. In KCMC, the ICU admission category was based mainly on infectious (20.5%) and cardiac (9%) diseases [7]. In this study, emergencies such as upper gastrointestinal bleeding and NCDs such as diabetic ketoacidosis and heart failure dominated the admission charts. The observed trend shows that developing countries have a dual burden of NCDs as well as communicable diseases. Previously, medical admissions were mainly for communicable diseases in developing countries, but now there is a shifting pattern of diseases toward NCDs, especially in the tropics [20].

## Conclusion

The overall mortality rate in the MICU was considerably high, with medical emergencies and cardiovascular disorders being the most common causes of admission and death. Elderly patients, transfers from the medical ward to the MICU, and short ICU stays were significantly associated with the poor outcome of MICU patients. Most critically ill patients died within 24 hours of admission. Therefore, further studies need to be conducted to understand the cause of the high mortality rate. This may help reduce the mortality rate among critically ill patients admitted to the MICU.
